# Intracranial Aneurysms and Cerebral Small Vessel Disease: Is There an Association between Large- and Small-Artery Diseases?

**DOI:** 10.3390/jcm13195864

**Published:** 2024-10-01

**Authors:** Vanessa M. Swiatek, Stefanie Schreiber, Amir Amini, David Hasan, Ali Rashidi, Klaus-Peter Stein, Belal Neyazi, I. Erol Sandalcioglu

**Affiliations:** 1 Department of Neurosurgery, Otto-von-Guericke University, 39120 Magdeburg, Germany; vanessa.swiatek@med.ovgu.de (V.M.S.); amir.amini@med.ovgu.de (A.A.); ali.rashidi@med.ovgu.de (A.R.); klaus-peter.stein@med.ovgu.de (K.-P.S.); erol.sandalcioglu@med.ovgu.de (I.E.S.); 2 Department of Neurology, Otto-von-Guericke University, 39120 Magdeburg, Germany; stefanie.schreiber@med.ovgu.de; 3 German Center for Neurodegenerative Diseases (DZNE), 39120 Magdeburg, Germany; 4 Center for Behavioral Brain Sciences, Otto-von-Guericke University, 39120 Magdeburg, Germany; 5 Department of Neurosurgery, Duke University, Durham, NC 27707, USA; david.hasan@duke.edu

**Keywords:** intracranial aneurysms, cerebral small-vessel disease, crosslink

## Abstract

**Background/Objectives**: Intracranial aneurysms (IAs) may be connected to interactions between large and small intracranial vessels. We aimed to investigate the association between IAs and cerebral small-vessel disease (CSVD) and assess CSVD impact on IA patient management. **Methods**: This retrospective study analyzed clinical data and MRI features of CSVD in 192 subarachnoid hemorrhage (SAH) patients: 136 with incidental IA, 147 with severe CSVD without SAH/IA, and 50 controls without SAH, IA, or severe CSVD. MRI assessments followed the Standards for Reporting Vascular Changes on Neuroimaging (STRIVE), with a total burden of small-vessel disease (TBSVD) score calculated. Statistical analyses included forward selection and binary logistic regression. **Results**: TBSVD differed significantly across groups (*p* < 0.001), except between SAH and IA groups (*p* = 0.8). Controls had the lowest TBSVD (1.00; 1.22 ± 0.996), followed by SAH (2.00; 2.08 ± 1.013) and IA groups (2.00; 2.04 ± 1.141), with the highest in the CSVD group (1.00; 1.22 ± 0.996). White-matter hyperintensity (WMH) patterns varied with IA rupture status (*p* = 0.044); type A was prevalent in SAH patients and type D in the IA group. Incorporating MRI CSVD features and TBSVD into risk assessments did not enhance IA prediction or outcome models. **Conclusions**: IA patients exhibit a higher CSVD burden than controls, suggesting a link between small and large intracranial vessels. WMH patterns distinguish between ruptured and unruptured IA patients, offering potential markers for IA rupture risk assessment and signaling a paradigm shift in understanding IAs and CSVD.

## 1. Introduction

Intracranial aneurysms (IAs) constitute a major challenge in the field of neurosurgery [1,2,3,4]. Unruptured IAs (UIAs) are present in 3–5% of the adult population worldwide, with no significant variation across different geographic regions or ethnic groups [2,5,6]. These aneurysms typically begin to form after the age of 20, with the peak incidence occurring between the ages of 40 and 60. Women are affected more frequently than men [2,5,7,8]. The estimated global incidence of subarachnoid hemorrhage (SAH) resulting from aneurysm rupture is about 6.7 cases per 100,000 people annually, leading to approximately 500,000 cases worldwide each year [9]. Several models to estimate the rupture risk of IA have been introduced. Aside from clinical scores that weight established morphological and clinical aspects of IA, more recent approaches have focused on inflammatory processes and imaging modalities for the assessment of rupture risk. 

IAs are regarded as large-vessel diseases [1], the counterpart of which is the group of cerebral small-vessel diseases (CSVDs) [10,11]. CSVD is a microvascular condition common in older adults and present in approximately 50% of those over 65 [12,13,14,15,16]. There are no significant gender differences [17,18] (Hilal, Cannistraro). It is a primary cause of lacunar strokes [12,18,19,20,21] and significantly contributes to vascular cognitive impairment and dementia [12,18,22,23,24]. CSVD frequently correlates with hypertension and is characterized, among other features, by arteriolosclerosis in cerebral small arteries [12,25].

Consideration of IAs as a pathology located in a continuous circulatory system would suggest an interconnection of different diseases affecting the small and large intracranial arteries [10,26]. In addition to anatomical proximity of the affected vascular segment, IAs and CSVDs share overlapping pathogenesis. Arterial hypertension, encompassing hemodynamic stress and the resulting damage to the endothelium, has a key role in IA and CSVD [10,27,28,29,30,31,32]. Endothelial damage leads to a complex signaling cascade of inflammatory processes and ultimately aneurysm wall remodeling in the large vessels [27] and disruption of the blood–brain barrier in the small vessels of the brain [10].

In the field of vascular surgery, the role of CSVD in the development of cognitive impairment after carotid revascularization is highly discussed [33,34,35,36]. Arba et al. proposed that stratification of patients based on CSVD prior to the operative procedure is likely to gain importance for proper treatment selection [35]. Taken together, these results imply a possible interconnection between vascular pathologies of the small and the large intracranial vessels, and this hypothesis suggests that pathologies of the cerebral vascular system should be referred to as diseases of the same continuous circulatory system [10]. Cardiovascular risk factors therefore cause pathological changes in all sections of the vascular system, which in turn can feed back to each other [26]. However, possible implications of interactions between small and large intracranial vessels and their pathologies on the morbidity and mortality of IA patients remains uninvestigated.

Here, we investigated the associations between IA and CSVD and their possible influence on future studies addressing the clinical management of IA patients. Retrospective clinical data and magnetic resonance imaging (MRI) features were evaluated in this context.

## 2. Materials and Methods

### 2.1. Study Population

This manuscript adheres to the guidelines for reporting observational studies [37]. The ethics committee of the Otto-von-Guericke University waivered the analysis of retrospectively collected data (Ethics Committee Vote No. 94/20, 146/19, and 28/16 and addendum No. 01/23). 

Patients with IAs and CSVD treated at the Departments of Neurosurgery and Neurology at Otto-von-Guericke University between 1996 and 2018 were included in the study. Additionally, a control cohort comprising patients without intracranial vascular diseases was established. All patients included in this study met the following inclusion criteria regardless of the primary disease:

1. MRI imaging of the neurocranium performed in the context of clinical diagnostics with T2-weighted sequences or T2 fluid-attenuated inversion recovery (FLAIR) and T2* sequences or susceptibility-weighted imaging (SWI);
2. Sufficient quality of MRI data for scoring of lesions related to CSVD;
3. Information on clinical parameters.

**Figure 1:**
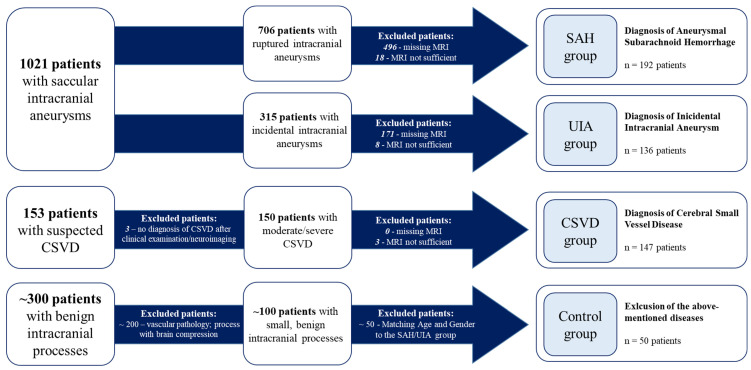
Categorization of the included patients with respect to the underlying disease. CSVD, cerebral small-vessel disease; UIA, unruptured intracranial aneurysm; SAH, subarachnoid hemorrhage.

Overall, 525 patients met the inclusion criteria and were categorized into four cohorts (SAH, UIA, CSVD, and control) as follows (Figure 1):

IA patients were divided into two groups: the UIA group for incidental cases and the SAH group for those with ruptured IAs (Figure 1). In some analyses, these two groups were combined and referred to as the IA group.

CSVD patients were admitted to the Department of Neurology and diagnosed with moderate to severe CSVD. Diagnosis was based on clinical presentation, including ischemic strokes attributable to CSVD, cognitive decline, dementia, psychiatric disorders, abnormal gait, and urinary incontinence [38], in conjunction with neuroimaging findings from MRI. The diagnostic process adhered to the Standards for Reporting Vascular Changes on Neuroimaging (STRIVE) criteria [11]. The presence of IAs was conclusively ruled out in this cohort (Figure 1).

In addition, a control group of patients without intracranial vascular diseases was established. Fifty patients with small, benign intracranial processes treated at the neurosurgical department of the Otto-von-Guericke University were analyzed according to the study protocol. These patients did not show evidence of intracranial vascular diseases (Figure 1). The diagnoses included in this cohort are shown in the Supplemental Material (Table S1). The control cohort was matched to the age and gender distribution of the SAH and UIA group to minimize confounding factors.

For clinical parameters, particular attention was paid to the inclusion of vascular risk factors and, for the SAH and UIA groups, of established parameters in the clinical assessment and treatment of these patients [39,40,41,42,43,44,45,46,47,48,49]. An overview of collected clinical parameters is shown in Table 1. Patient characteristics of all four cohorts and IA attributes are shown in Table 2.

### 2.2. MRI Acquisition

MRI was performed using 1 Tesla (n = 24; 5%), 1.5 Tesla (n = 305; 58%), and 3 Tesla (n = 196; 37%) scanners. The included sequences were either T2-weighted sequences (2 to 6 mm slice thickness; 2404 to 11,122 millisecond (ms) repetition time (RT); 15 to 143 ms echo time (ET)) or FLAIR (2 to 6 mm slice thickness; 4800 to 12,000 ms RT; 77 to 307 ms ET), T2* sequences (3 to 6 mm slice thickness; 340 to 11,978 ms RT; 4 to 80 ms ET), or SWI (1.5 to 8 mm slice thickness; 23 to 75 ms RT; 0 to 48 ms ET).

### 2.3. MRI Analysis

MRI analysis of all four cohorts was carried out in a semi-quantitative manner in accordance with the STRIVE [11]. The investigator was not blinded to the allocation of patients to individual cohorts. MRI images were examined using the CHILI PACS platform (NEXUS/CHILI GmbH; version 4.38.2, build 23; 2018) and in compliance with specific methods and scales (see below). 

White-matter hyperintensities (WMHs) of presumed vascular origin are associated with cerebrovascular diseases and vascular risk factors; however, the pathogenesis is considered to be multifactorial [16,50]. WMHs were rated using axial T2 FLAIR images (n = 401), coronary T2 FLAIR images (n = 54), or, in case of missing FLAIR sequences, in axial T2-weighted images (n = 70). 

**Figure 2:**
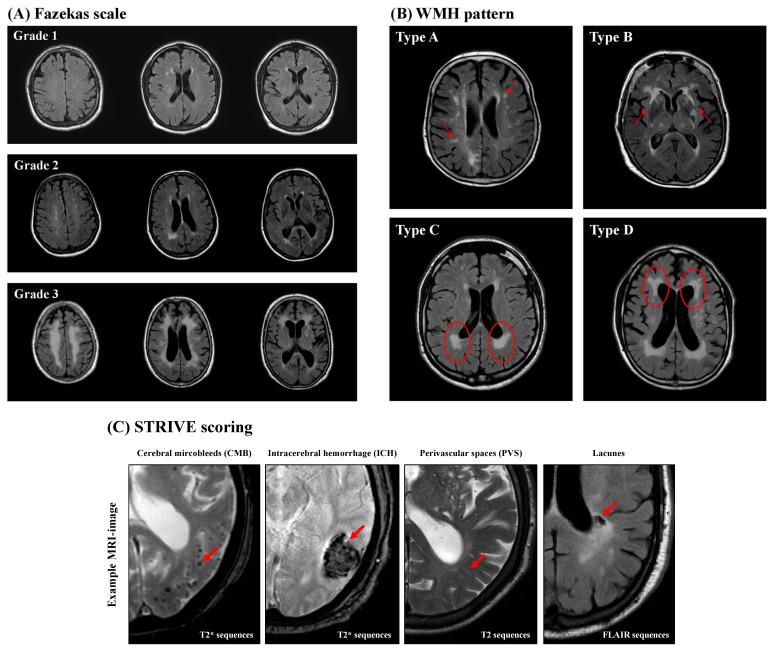
Representative magnetic resonance imaging (MRI). The Fazekas scale (**A**) is a graduated scoring system in which the degree of white-matter hyperintensity (WMH) increases with the degree of WMH (grades 1–3). The WMH pattern (**B**) categorizes the distribution of WMH in a quantitative way into four types: multiple subcortical spots (type A), peri-basal ganglia WMH (type B), posterior subcortical pattern (type C), and anterior subcortical pattern (type D). The red circles and red arrows mark the characteristic MRI findings. (**C**) STRIVE MRI scoring. The red arrows mark the characteristic MRI findings.

WMH analysis was performed in accordance to the Fazekas scale (grades 1–3), which separates periventricular white matter and deep white matter for the three separate cerebral hemispheres [51], and the WMH pattern introduced by Charidimou et al. [52] (Figure 2). Charidimou et al. defined four WMH patterns in patients suffering from two different types of CSVD (cerebral amyloid angiopathy (CAA) and hypertensive arteriopathy (HA)): multiple subcortical spots (type A), peri-basal ganglia WMH (type B), posterior subcortical pattern (type C), and anterior subcortical pattern (type D). If there was no evidence of WMH detected in the examined patient, a score of zero was assigned for both classifications.

Cerebral microbleeds (CMBs) are small round- or oval-shaped lesions with a diameter of 2–10 mm and have hypointense appearance in axial (n = 479) or coronary (n = 34) T2* sequences or axial SWI (n = 12). CMBs are not visible in T2 FLAIR or T1- or T2-weighted sequences (Figure 2). The Microbleed Anatomical Rating Scale (MARS) was used to further categorize CMBs into the following three groups of anatomical regions: lobar (frontal, temporal, parietal, occipital, and insula); deep (basal ganglia, thalamus, internal capsule, external capsule, corpus callosum, deep and periventricular white matter, and brainstem); and infratentorial (cerebellum) [11,53,54]. The total CMB count was also determined for the whole brain, and a CMB ratio was calculated by dividing the count of lobar CMBs by the number of deep CMBs [55].

Intracerebral hemorrhages (ICHs) are larger lesions (>10 mm) that appear in axial T2* sequences (n = 479), coronary T2* sequences (n = 34), or axial SWI (n = 12), similarly to CMBs (Figure 2). They were classified according to the Cerebral Hemorrhage Anatomical Rating Instruments (CHARTS) into the same three anatomical categories described for CMB. The anatomical region (lobar, deep, or infratentorial) was determined using the largest diameter and epicenter of the ICH. The ICH ratio was calculated in a similar fashion as the CMB ratio [11,56,57].

Perivascular spaces (PVSs) are fluid-filled spaces surrounding small vessels of the brain and appear in MRI as small, elongated striations with a maximum diameter of 3 mm and a cerebrospinal fluid-like signaling on T2 FLAIR and T2-weighted images without a surrounding hyperintense rim [58] (Figure 2). The severity of PVS was determined using axial T2-weighted (n = 412) or axial T2 FLAIR (n = 113) imaging and counted separately in the centrum semiovale (CSO), defined as the planes above the lateral ventricle and corpus callosum, as well as the basal ganglia (BG), defined as the caudate nucleus, internal capsule, thalamus, lentiform nucleus, external/extreme capsules, and insular cortex [59]. At least three MRI slices were examined to determine the number of PVSs in the CSO and BG, whereby both hemispheres of the brain were examined, followed by definition and analysis of the hemisphere with the larger PVS burden. Finally, CSO- and BG-PVS were classified separately as either mild (<10 PVSs), moderate (11–20 PVSs), frequent (21–40 PVSs), or severe (>40 PVSs) [59,60]. Based on the above-mentioned evaluation of the PVSs, a further classification into three subtypes was carried out: CSO-PVS-predominant type; BG-PVS-predominant type; and an equal type with similar distribution of PVSs in the CSO and the BG [61]. 

Lacunes are assumed to be caused by small subcortical infarcts or deep hemorrhages [62]. They are defined as small (diameter 3–15 mm), hypointense-appearing lesions in T2 FLAIR sequences with a characteristic surrounding hyperintense rim (Figure 2). They were rated in axial (n = 401) or coronary (n = 53) T2 FLAIR sequences or axial T2 weighted-sequences (n = 71) [11] and subsequently categorized into the same three anatomical groups as described in the CMB and ICH sections according to MARS. Further analysis of the total lacunes count and the lacunes ratio was performed as described for CMBs.

To illustrate the manifestation of CSVD more clearly by MRI, Klarenbeek et al. developed a scale incorporating four of the above-mentioned MRI features of CSVD, with each feature being awarded one point (range 0–4). This total burden of small-vessel disease (TBSVD) score more accurately reflects the overall effect of CSVD compared to individual features and therefore better represents overall brain damage [63]. 

The TBSVD score was calculated based on four CSVD-related criteria according to the score introduced by Klarenbeek et al.: presence of lacunes (≥1); CMBs (≥1 microbleeds); PVS (moderate (11–20) to severe (>40) BG burden); and WMH (Fazekas scale measured in the periventricular white matter measured as grade 3; and/or Fazekas scale measured in the deep white matter as grade 2–3) [63,64]. 

In a previously published study examining SAH patients with vasospasm using combined diffusion-weighted and hemodynamically weighted MRI, results showed hemodynamic abnormalities in regions with confirmed vasospasm. These MRI changes, however, did not influence the CSVD scoring in SAH patients. This indicates that while vasospasms can alter cerebral hemodynamics, they do not directly correlate with CSVD scores [65,66]. On the other hand, a recently published review exploring the role of MRI imaging in SAH patients unveiled that SAH and IA treatment for ruptured IA might cause distinct MRI abnormalities, thereby potentially impacting CSVD scoring [67]. Nonetheless, in this investigation, meticulous analysis of MRI timing and the selected treatment modality for IA treatment, with respect to their impact on the TBSVD score, revealed no statistical disparities, suggesting that these variables did not systematically influence the MRI scoring.

### 2.4. Statistical Analysis

Statistical analysis was performed using SPSS Statistics for Windows (IBM^®^ Corp. Released 2021. IBM SPSS Statistics for Windows, Version 28.0. Armonk, NY, USA: IBM Corp. version 28.0). The significance level was set at *p* ≤ 0.05. If necessary, a Bonferroni correction was applied. Normal distribution of data was tested by the Kolmogorov–Smirnov test. Due to more than two cohorts being tested against each other, the non-parametric Kruskal–Wallis test was applied. To further interpret the results of the Kruskal–Wallis test, several consecutive Mann–Whitney U-tests were conducted to compare the four cohorts in case of significant results. Nominal scaled parameters were analyzed by means of cross-tables and subsequent determination of the chi-square and standardized residuals. If necessary, a Fisher correction was applied. 

Following univariate calculations, multivariate analyses of the relevant parameters were conducted using binary logistic hierarchical regressions with inclusion of parameters in defined blocks. The outcome parameters addressed in the multivariate analyses were identification of patients with multiple IAs, prediction of aneurysm rupture, and clinical outcome of patients (modified Rankin scale (mRS) at the time of discharge, dichotomized into the groups “favorable outcome” (mRS = 0–3) and “unfavorable outcome” (mRS = 4–6)) with IAs.

## 3. Results

### 3.1. TBSVD

**Figure 3:**
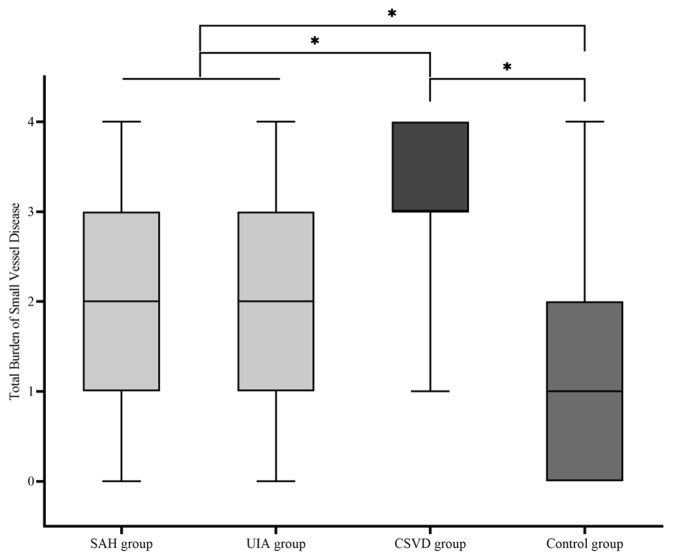
Boxplot of the total burden of small-vessel disease (TBSVD) scores for the four cohorts. CSVD, cerebral small-vessel disease; UIA, unruptured intracranial aneurysm; SAH, subarachnoid hemorrhage. * statistical significance.

Descriptive statistical analysis was used to test whether there was a difference between the four cohorts in terms of the TBSVD as a summative score of typical MRI features (median; mean ± standard deviation): SAH group (2.00; 2.08 ± 1.013), UIA group (2.00; 2.04 ± 1.141), CSVD group (3.00; 3.17 ± 0.797), and control group (1.00; 1.22 ± 0.996) (Figure 3). A comparative analysis of the four cohorts showed a significant difference of TBSVD scores between all cohorts (*p* < 0.001), except for the comparison of the SAH group against the UIA group (*p* = 0.8). Among these, the control group showed the lowest TBSVD, the SAH and UIA groups a medium TBSVD, and the CSVD group the highest TBSVD.

### 3.2. WMH Pattern

**Figure 4:**
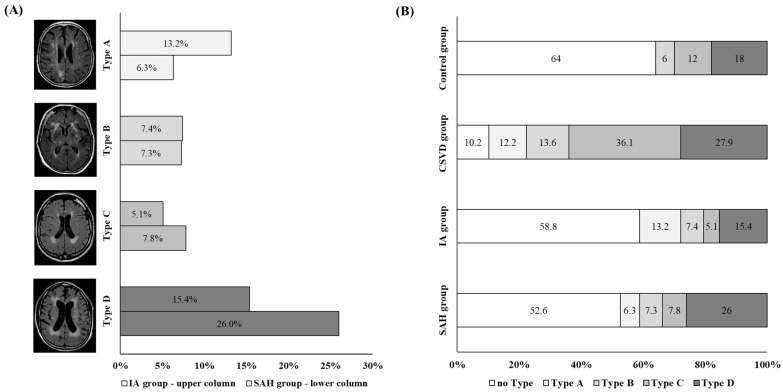
(**A**) Illustration of the distribution of white-matter hyperintensity (WMH) pattern types in patients with SAH (SAH group; upper bar for each type) and UIA (UIA group; lower bar for each type) groups. We found that type A was more common in the UIA group (represented by light-grey bars), while type D was more frequent in the SAH group (represented by dark-grey bars). (**B**) Illustration of the distribution of WMH pattern. The proportion of WMH pattern within the cohorts is shown within the columns as a percentage. The light-grey-colored columns highlight type A, while the dark-grey-colored columns highlight type D.

The WMH patterns in the individual cohorts were unequally distributed (*p* < 0.001) (Figure 4). Type A was underrepresented in the control group, while type C was underrepresented in the SAH and UIA groups. Type C was the only pattern overrepresented in the CSVD group.

Further analyses were performed to determine whether the WMH pattern varied in patients with IAs depending on aneurysm localization, size, and rupture status. For this analysis, the SAH group and the UIA group were combined into one group, which was then referred to as the IA group. No difference in WMH pattern was observed when comparing the localization of the IA within the anterior against the posterior circulation (*p* = 0.182) or with respect to the size of the aneurysm (*p* = 0.251). However, the distribution of the WMH pattern in relation to the rupture status of the aneurysm was significantly different (*p* = 0.044). Here, an overrepresentation of type D in patients with ruptured IAs and an overrepresentation of type A in patients with UIAs were found (Figure 4).

### 3.3. Investigation of IA Patients

To determine the impact of CSVD on the clinical management of IA patients, a multivariate analysis of CSVD MRI features on IA multiplicity, IA rupture, and the clinical outcome of IA patients was performed. 

First, univariate analyses were conducted to identify significant parameters. The subsequent multivariate analysis was performed by means of a binary logistic hierarchical regression with inclusion of parameters in defined blocks. Hence, all parameters found to be significant in the univariate analysis were included in the final regression models. The included parameters, however, contributed to the prediction of the regression model to different extents.

#### 3.3.1. Aneurysm Multiplicity

Analysis of the clinical and MRI parameters in relation to comparison of patients with singular and multiple aneurysms was performed for the IA group (SAH and UIA groups together). Here, patients were more likely to be female (*p* < 0.001) and tended to suffer less from ischemic stroke (*p* = 0.070). The WMH pattern type D (*p* = 0.067) and the BG type of PVS (*p* = 0.086) tended to appear more often in patients with multiple IAs. Due to the low number of significant parameters in this analysis, no further multivariate analysis was conducted.

#### 3.3.2. Aneurysm Rupture

We tested whether the clinical and MRI parameters differed between ruptured and unruptured aneurysms, i.e., between the SAH and UIA groups. Seven clinical parameters, including younger age and smaller size in SAH patients along with five MRI parameters, were different between the SAH and UIA groups (Table S2). Within the final regression model, the following parameters were significantly involved in predicting aneurysm rupture: lower age at diagnosis (*p* < 0.001), less frequent presence of stroke (*p* = 0.004), smaller aneurysm size (*p* = 0.001), and higher number of ICHs (*p* = 0.010) (Table S2). The percentage of patients correctly classified by the model as having a ruptured or unruptured aneurysm was 75.6%.

#### 3.3.3. Clinical Outcome of Aneurysm Patients

To first conduct a general statistical analysis of the clinical outcome, parameters were identified that showed differences between patients with favorable or unfavorable outcomes for the SAH and UIA groups together. Here, four clinical and seven MRI parameters varied between IA patients (Table S3). Patients with aneurysm rupture, low Glasgow coma scale (GCS) scores on admission, and occurrence of a post-hemorrhagic hydrocephalus were more likely to have an unfavorable outcome. 

For the final model predicting unfavorable outcomes for the IA group (SAH and UIA groups together), the following parameters contributed the most to model prediction: more frequent presence of hypertension (*p* = 0.048), aneurysm rupture (*p* = 0.042), and lower GCS at admission (*p* < 0.001). In this model, the MRI parameters failed to provide a contribution to the predictive value (Table S3). The percentage of patients correctly classified by the model as having a favorable or unfavorable outcome was 91.6%. 

The outcome of patients with SAH was studied separately due to their higher probability of unfavorable outcome compared to patients with unruptured IA. Twelve clinical parameters and eight MRI parameters showed differences between SAH patients with favorable and unfavorable outcomes (Table S3). More frequent presence of hypertension (*p* = 0.042) and a higher World Federation of Neurological Surgeons (WFNS) score at admission (*p* = 0.004) were the significant parameters that contributed to prediction of rupture status. In this model, MRI parameters did not contribute to the predictive value (Table S3). The percentage of patients correctly classified by the model as having a favorable or unfavorable outcome was 89.7%. Only a small number of patients suffered an unfavorable outcome in the UIA group; therefore, no complementary investigation of this cohort was performed.

## 4. Discussion

IA can be classified as a large-vessel disease of the brain. CSVD, however, is a generic term for severe diseases affecting the small intracranial vessels. Thus far, IA and CSVD have been analyzed as separate vascular brain diseases. Several studies have addressed the interconnection of either IA or CSVD with other intra- or extracranial vascular diseases and vascular risk factors. To our knowledge, however, the association between these two pathologies has not been investigated. Therefore, the aim of this study was to investigate MRI features of CSVD in patients with ruptured and unruptured IA. For validation of our results, we implemented a control group consisting of patients without evidence of intracranial vascular diseases. 

The results of this study showed that patients with IA had a higher TBSVD than the control group. Several studies have examined the association between pathological changes of large extracranial vessels and imaging features of CSVD [33,34,35]. For example, carotid atherosclerosis is a common disease of extracranial large vessels and has an increased prevalence of MRI features. Furthermore, the comorbidity of systemic atherosclerotic disease and evidence of CSVD on MRI indicates a higher risk of death and ischemic stroke in these patients [33,34,35]. 

Several vascular risk factors are associated with the pathogenesis of IAs. The formation [5,68] and subsequent rupture of IA is linked to arterial hypertension [40,69,70], smoking [40,69,70,71], heavy alcohol consumption [40,72,73], and sleep apnea syndrome [74]. Protective factors that are thought to reduce the risk of rupture include physical activity [75,76,77] and, paradoxically, a high body mass index [70,78], diabetes [40], and hypercholesterolemia [40,79].

In contrast, risk factors for CSVD include advanced age [18,80,81], arterial hypertension [17,18], nicotine abuse [18,82], diabetes [82], sleep apnea syndrome [83], chronic kidney disease [84], and previous strokes [85]. Common risk factors for both conditions are therefore arterial hypertension, nicotine abuse, and sleep apnea syndrome. While hypercholesterolemia is a risk factor for large vessel disease, its impact on the risk of CSVD in the current population is difficult to assess due to the widespread use of statin medications [86]. Similarly, the data regarding its influence on the formation and rupture of aneurysms are paradoxical, with some studies suggesting a protective effect [40,79].

Although diabetes is a risk factor for small-vessel damage and the development of CSVD [82], current evidence suggests that, paradoxically, it may have a protective effect against IA formation and rupture [40]. The overlap of some risk factors clearly supports the hypothesis that large-vessel diseases of the brain may coexist with small-vessel disease. On the other hand, the differing effects of certain risk factors may lead to significant differences between the two conditions. These similarities and differences warrant further investigation, particularly in prospective studies.

In our study, analysis of WMH patterns showed that ruptured IAs present more often with anterior subcortical patches (type D), while unruptured IAs show predominantly multiple subcortical spot pattern (WMH pattern type A). Four different WMH patterns were described by Charidimou et al. [52]. These patterns were investigated in relation to CSVD subgroups CAA and HA. Multiple subcortical spots (type A) were found to be more common in CAA, while the peri-BG WMH pattern (type B) showed associations with HA diagnosis. The large posterior subcortical patches pattern (type C) and the large anterior subcortical patches (type D) are not associated with any specific cerebrovascular disease [52]. Future studies should therefore focus on the clinical relevance of these WMH patterns not only for patients with intracerebral hemorrhage but with other intracranial vascular pathologies as well.

Based on previous findings, we aimed to investigate whether the addition of MRI features of CSVD to established clinical parameters might contribute to a better prediction model for clinical outcomes, with the goal of enhancing management of IA patients. In clinical practice, aneurysm rupture, the presence of multiple IAs, and the clinical outcome of IA patients are of key importance. Our results show that MRI features of CSVD do not contribute in a substantial extent to the prediction of these three clinical factors. 

It is important to mention factors that could be considered as possible limitations of this study. First, this study was conducted based on retrospectively collected data. Due to the retrospective design of this study, MRI imaging was obtained from clinical diagnostics and not performed in a standardized manner. The type of MRI scanner and the MRI field strength as well as the evaluated MRI sectional plane and sequences were not included as co-variables into the statistical analysis. Second, MRI scoring was performed by only one rater. However, MRI scoring was conducted in a standardized format in strict accordance with the guidelines reported by the STRIVE protocol [11]. Third, there is a clear survivorship bias, particularly for the SAH group, as patients who did not survive the acute phase of SAH were not included in the study due to the lack of MRI performed in those cases. This may have impacted the results and could influence the interpretation of our findings. The exclusion of these patients who might have had more severe conditions could result in a skewed understanding of outcomes and patient characteristics. Additionally, the vascular risk factors between IA patients and the control group were not matched, which represents a limitation of our study. As a result, our findings should not be generalized, especially when comparing the burden of CSVD in IA patients to those with extracranial vascular diseases.

## 5. Conclusions

In summary, patients with IA presented with a higher burden of small intracranial vessel damage compared to a vascularly healthy control group. However, a comparison between ruptured and unruptured IA did not show a difference in the severity of MRI features related to CSVD. Regarding WMH distribution patterns, a more frequent occurrence of anterior subcortical patches in ruptured IA and multiple subcortical spot patterns in unruptured IA could be demonstrated. Future studies should investigate the crosslink between IA and CSVD in a prospective study design to minimize confounding factors and to further illuminate actual pathophysiological relationships and their clinical significance.

## Figures and Tables

**Table 1:** Overview of clinical parameters [39,40,41,42,43,44,45,46,47,48,49].

Parameter Category	Parameters/Definitions	Included Cohort
Epidemiological data	Age (defined as age at diagnosis)	1–4
	Gender (defined as biological gender)	1–4
Medical history	Hypertension (defined as documented diagnosis or intake of antihypertensive medication)	1–4
	Diabetes mellitus (defined as documented diagnosis or type 1 or 2 diabetes or intake of oral antidiabetics or insulin)	1–4
	Hyperlipidemia (defined as documented diagnosis or intake of medication lowering the lipid or cholesterol levels)	1–4
	Peripheral arterial disease (defined as documented diagnosis or imaging finding)	1–4
	Heart disease (defined as documented diagnosis of myocardial infarction, coronary artery, disease, cardiac arrhythmia, or other heart diseases)	1–4
	Ischemic stroke (defined as documented diagnosis or imaging finding at admission)	1–4
	Thrombosis (defined as documented diagnosis)	1–4
	Benign or malignant tumor disease (defined as documented diagnosis regardless of affected organ)	1–4
	Autoimmune disease (defined as documented diagnosis with need of immunosuppressive therapy)	1–4
	Obesity (defined as documented body mass index of >30 kg/m^2^)	1–4
	Nicotine abuse (defined as ex-nicotine abuse or continued nicotine abuse)	1–4
	Alcohol abuse (defined as consumption of >50 g of alcohol per week)	1–4
	Contraceptive use (at time of diagnosis, extracted from the medical records or medication plan)	1–4
CT-imaging parameters	Type of bleeding, shifting of the midline, intraventricular hemorrhage, hydrocephalus and isch emia (in the first CT scan after SAH)	1
Aneurysm-related parameters	Rupture status (defined by assessment of intraoperative findings, imaging findings, and bleeding patterns in CT)	1 + 2
	Multiplicity (defined as ≥2 intracranial aneurysms) and number of aneurysms	1 + 2
	Aneurysm localization (defined by angiography results)	1 + 2
	Size of the aneurysm (defined by measuring the maximum diameter in 2D angiography)	1 + 2
Clinical scores	Glasgow coma scale at admission and discharge (defined by neurological examination at admission and discharge)	1 + 2
	Modified Rankin scale and outcome at discharge (defined by neurological examination at discharge)	1 + 2
	Hunt and Hess grade, WFNS score, and Fisher grade at admission (defined by neurological examination at admission and imaging)	1
	PHASES score (defined by use of clinical data and examination of neuroradiological findings)	1 + 2
Treatment-related parameters	Previous and current treatment and treatment modality (extracted from medical records)	1 + 2
Complication-related parameters	Presence of hydrocephalus, placement of external ventricular drainage, or ventriculoperitoneal shunt (extracted from medical records)	1 + 2
	Presence of vasospasm, method of vasospasm-detection, and treatment via endovascular spasmolysis (extracted from medical records)	1 + 2
Follow-up data	Time of follow-up, perfusion of the aneurysm, and modified Rankin scale and outcome at follow-up (extracted from medical records)	1 + 2

SAH, subarachnoid hemorrhage; CT, computed tomography.

**Table 2:** Patient characteristics.

	Cohort 1 (SAH Group) n = 192	Cohort 2 (UIA Group) n = 136	Cohort 3 (CSVD Group) n = 147	Cohort 4 (Control Group) n = 50	Total Cohort n = 525	General Statistics
Gender distribution (n)						*p* < 0.001 **
Male	53 (27.6%)	38 (27.9%)	76 (51.7%)	14 (28%)	181 (35%)	
Female	139 (72.4%)	98 (72.1%)	71 (48.3%)	36 (72%)	344 (65%)	
Mean age (years)	50.03	59.38	71.21	57.28	59.07	*p* < 0.001 *
Medical history (n)						
Hypertension	126 (66%)	100 (74%)	130 (88%)	29 (58%)	385 (73%)	*p* < 0.001 **
Diabetes	25 (13%)	22 (16%)	34 (23%)	6 (12%)	87 (17%)	*p* < 0.001 **
Hyperlipidemia	41 (21%)	52 (38%)	94 (64%)	9 (18%)	196 (37%)	*p* < 0.001 **
Peripheral arterial disease	5 (3%)	3 (2%)	6 (4%)	0 (0%)	14 (3%)	*p* = 0.408 **
Heart disease	23 (12%)	42 (31%)	68 (46%)	13 (26%)	146 (28%)	*p* < 0.001 ***
Ischemic stroke	7 (4%)	28 (21%)	64 (44%)	1 (2%)	100 (19%)	*p* < 0.001 **
Thrombosis	9 (5%)	8 (6%)	6 (4%)	4 (8%)	27 (5%)	*p* = 0.743 **
Tumor disease	16 (8%)	32 (24%)	39 (27%)	12 (24%)	99 (19%)	*p* < 0.001 **
Autoimmune disease	9 (5%)	11 (8%)	13 (9%)	2 (4%)	35 (7%)	*p* = 0.298 **
Lifestyle and Medication (n)						
Nicotine abuse	95 (49%)	63 (46%)	41 (28%)	15 (28%)	214 (41%)	*p* < 0.001 **
Alcohol abuse	26 (14%)	19 (14%)	29 (20%)	4 (8%)	78 (15%)	*p* < 0.001 **
Obesity	52 (27%)	43 (32%)	37 (25%)	15 (30%)	147 (28%)	*p* = 0.910 **
Contraceptive use	6 (3%)	5 (4%)	0 (0%)	0 (0%)	11 (2%)	*p* = 0.031 ***
Mean aneurysm size (mm)	7	9				*p* < 0.001 ****
Aneurysm multiplicity	64 (33%)	36 (26%)				*p* = 0.183 **

CSVD, cerebral small-vessel disease; UIA, unruptured intracranial aneurysm; SAH, subarachnoid hemorrhage; * Kruskal–Wallis test; ** chi-square test; *** chi-square test with Fisher correction; **** Mann–Whitney-U test. Significant results are highlighted in bold.

## Data Availability

The data supporting the findings of this study are not openly available due to reasons of sensitivity and are available from the corresponding author upon reasonable request.

## Supplementary Materials

The following supporting information can be downloaded at https://www.mdpi.com/article/10.3390/jcm13195864/s1, Table S1: Overview of all collected parameters in this study for all four cohorts, including data extracted from MRI scoring; Table S2: Presentation of the results of the univariate and multivariate analysis regarding the rupture status of IAs with indication of the significances (p) and the regression coefficients (b). The final model classified the patients correctly in 75.6% of the cases; Table S3: Presentation of the results of the univariate and multivariate analysis regarding the clinical outcome of patients with IAs separated in all aneurysms and ruptured aneurysms with indication of the significances (p) and the regression coefficients (b). The final model for all patients with IAs classified the patients correctly in 91% of the cases; the final model for patients with ruptured IAs classified the patients correctly in 89.7% of the cases.
