# Supplementary material for: Intracranial Aneurysms and Cerebral Small Vessel Disease: Is There an Association between Large- and Small-Artery Diseases?
Source: J Clin Med. 2024 Oct 1;13(19):5864. doi: 10.3390/jcm13195864 (PMC11476928; doi:10.3390/jcm13195864)
Supplement: Supplementary file 1 [file jcm-13-05864-s001.zip › Supplemental_CSVD/Table S2.pdf]

Aneurysm rupture	Univariat analysis	Multivariat analysis	
	p	b	p
**Age at diagnosis**	< 0.001	-0.095	**< 0.001**
**Cardiac disease**	< 0.001	-0.399	0.321
**Benign and malignant tumor diseases**	< 0.001	-0.79	0.073
**Hyperlipidämia**	0.001	-0.089	0.805
**Ischemic stroke**	< 0.001	-1.606	**0.004**
**Aneurysm size**	< 0.001	-0.106	**0.001**
**Fazekas scale (Periventricular right)**	*0.071*	0.05	0.949
**Fazekas scale (Periventricular left)**	*0.087*	0.683	0.38
**WMH pattern**	0.044	–	0.751
Type A	–	-0.221	0.69
Type B	–	0.415	0.555
Type C	–	0.279	0.701
Type D	–	0.542	0.307
**CMB total count**	0.022	0.002	0.982
**CMB ratio**	0.02	-0.146	0.24
**Lacunes total number**	0.04	-0.139	0.227
**PVS basal ganglia**	*0.071*	0.754	0.126
**PVS total number**	*0.09*	-0.24	0.382
**ICH total number**	< 0.001	3.639	**0.01**

Table S2: *Presentation of the results of the univariate and multivariate analysis regarding the rupture status of IAs with indication of the significances (p) and the regression coefficients (b). The final model classified the patients correctly in 75.6% of the cases.*
