# Supplementary material for: Intracranial Aneurysms and Cerebral Small Vessel Disease: Is There an Association between Large- and Small-Artery Diseases?
Source: J Clin Med. 2024 Oct 1;13(19):5864. doi: 10.3390/jcm13195864 (PMC11476928; doi:10.3390/jcm13195864)
Supplement: Supplementary file 1 [file jcm-13-05864-s001.zip › Supplemental_CSVD/Table S3.pdf]

Outcome SAH and IA group	Univariat analysis	Multivariat analysis	
	p	b	p
**Hypertension**	*0.062*	1.314	**0.048**
**Thrombosis**	0.008	1.769	0.076
**Aneurysm rupture**	< 0.001	1.717	**0.042**
**GCS at admission**	< 0.001	-0.364	**< 0.001**
**Therapy**	*0.06*	–	0.294
Surgical treatment	–	-1.093	0.471
Endovascular treatment	–	-0.403	0.762
Multimodal treatment	–	-2.460	0.14
**Posthemorrhagic hydrocephalus**	< 0.001	1.034	0.089
**Fazekas scale (Periventricular right)**	< 0.001	0.615	0.593
**Fazekas scale (Deep right)**	< 0.001	0.071	0.957
**Fazekas scale (Periventricular left)**	< 0.001	0.931	0.484
**Fazekas scale (Deep left)**	< 0.001	0.610	0.682
**WMH pattern**	< 0.001	–	0.799
Type A	–	-0.312	0.811
Type B	–	0.937	0.353
Type C	–	0.279	0.797
Type D	–	0.676	0.418
**ICH total number**	< 0.001	-0.592	0.362
**Total Burden of CSVD**	< 0.001	0.031	0.914

Outcome SAH group	Univariat analysis	Multivariat analysis	
	p	b	p
**Age at diagnosis**	0.014	0.024	0.471
**Hypertension**	0.031	1.718	**0.042**
**Diabetes**	0.019	0.242	0.668
**Thrombosis**	0.01	3.721	0.078
**Intraventricular hemorrhage on initial**	0.003	0.451	0.758
**Hydrocephalus on initial CT**	< 0.001	-0.659	0.511
**Hunt and Hess grade at admission**	< 0.001	-0.115	0.862
**GCS at admission**	< 0.001	0.437	0.109
**WFNS score at admission**	< 0.001	2.415	**0.004**
**Fisher grade at admission**	< 0.001	-0.45	0.737
**Type of bleeding on initial CT**	0.009	–	0.796
SAH + ICH	–	-0.907	0.499
ICH	–	20.076	1,000
**Posthemorrhagic hydrocephalus**	< 0.001	0.93	0.298
**Fazekas scale (Periventricular right)**	< 0.001	0.55	0.706
**Fazekas scale (Deep right)**	< 0.001	0.026	0.988
**Fazekas scale (Periventricular left)**	< 0.001	-1.204	0.586
**Fazekas scale (Deep left)**	< 0.001	1.298	0.621
**WMH pattern**	< 0.001	–	0.516
Type A	–	0.293	0.866
Type B	–	2	0.201
Type C	–	0.588	0.699
Type D	–	-0.237	0.848
**PVS total number**	0.043	-0.298	0.316
**ICH total number**	0.032	-0.025	0.98
**Total Burden of CSVD**	0.002	-0.26	0.553

Table S3: *Presentation of the results of the univariate and multivariate analysis regarding the clinical outcome of patients with IAs separated in all aneurysms and ruptured aneurysms with*

indication of the significances (p) and the regression coefficients (b). The final model for all patients with IAs classified the patients correctly in 91% of the cases; the final model for patients with ruptured IAs classified the patients correctly in 89.7% of the cases.
